# Little evidence that androstadienone affects social distance-dependent prosocial behaviour: a pre-registered study

**DOI:** 10.1098/rsos.240004

**Published:** 2024-05-22

**Authors:** Junsong Lu, Yuting Ye, Yin Wu

**Affiliations:** ^1^ Department of Applied Social Sciences, The Hong Kong Polytechnic University, Hung Hom, Kowloon, Hong Kong; ^2^ Institute of Psychology, School of Public Affairs, Xiamen University, Xiamen, People's Republic of China; ^3^ Research Institute for Sports Science and Technology, The Hong Kong Polytechnic University, Hung Hom, Kowloon, Hong Kong

**Keywords:** chemosignalling, androstadienone, prosocial behaviour, generosity, social distance

## Abstract

In navigating the complexities of social life, humans have evolved to interpret invisible odorous chemical cues, with profound behavioural impacts often unbeknown to the conscious mind. The manifestation of this in humans is evident in the scent of androstadienone (androsta-4,16-dien-3-one), an odorous compound which is considered a putative human pheromone. The current study investigated the effect of androstadienone on social distance-dependent prosocial behaviour measured by a social discounting task, in which participants chose between selfish and generous options. Based on our pre-registration, we predicted a sex-specific effect, with males exposed to androstadienone exhibiting increased generosity, while females would choose more selfishly. Employing a double-blind, placebo-controlled, between-subject design, we recruited 170 participants who were randomly assigned to either the androstadienone or control condition. Olfactory stimuli were administered while participants completed the social discounting task. Inconsistent with our hypothesis, inhaling androstadienone did not impact social distance-dependent prosocial behaviour. This finding was supported by multiple estimates of prosociality, including model-free, model-based and maximum likelihood estimation. Further analyses indicated that androstadienone administration did not influence perceived social distance or bias participants towards being generous or selfish. Thus, our empirical findings provide no support for the hypothesis that androstadienone modulates generosity.

## 1. Introduction

In countless social interactions, animals have developed sophisticated tools to navigate the social world, a crucial part of which includes interpreting chemosensory signals. The intricate exchange of chemicals signals various information, fine-tuning the cognitive and behavioural responses of the organism to effectively engage with its surrounding environment. Among the chemical senses, olfaction is central and has been suggested to transmit social signals, such as dominance, in animals [1]. Evidence for such communication has extended to humans more recently, refuting the long-standing misconception about the inferiority of the human sense of smell [2] and the insignificance of odorous signals in mediating human behaviours [3]. As noted by Lübke and Pause [3], the human axillary organ is exceptionally equipped for the production of odorous molecules, and numerous systems responsible for chemosignal transduction have evolved.

Previous studies have suggested that androstadienone (androsta-4,16-dien-3-one), one of the putative human sex pheromones [4,5], plays an important role in mediating social behaviour. It is an odorous compound emitted predominantly by males in axillary secretions and semen [6,7], which can stimulate sympathetic arousal and elevate cortisol levels [8,9]. Androstadienone has been associated with various roles in shaping a broad range of cognition and behaviour. It has been associated with increased attention to emotionally significant information [10] and with elevated mood and sexual arousal [9]. In social contexts, androstadienone modulates human aggression [11] and non-physical forms of aggression, such as the propensity to reject unfair offers in the ultimatum game [12]. To broaden our understanding of androstadienone in shaping social behaviour, the current study explored its influence on prosocial behaviour, a realm hitherto relatively unexplored. Prosocial behaviour overtly or covertly promotes the well-being of another individual or group, often at a cost to oneself, and is a vital component of a functioning society [13].

Although this area of research is still in its infancy, the expected link between androstadienone and prosocial behaviour can be derived from existing literature. Androstadienone has been regarded as a signal of male mate quality and dominance [14]. Specifically, it conveys information regarding potential mates, thus potentially affecting females’ preference for masculine face shapes, as well as increasing their perception of male attractiveness when exposed to it [4,15]. Since perceived attractiveness and dominance are known to shape cooperative behaviour [16,17], it is reasonable to hypothesize that androstadienone may also influence prosocial behaviour by altering social cognition towards others.

The potential influence of androstadienone on prosocial behaviour is hypothesized to be sex-specific. A common thread in the literature is that androstadienone affects a spectrum of social behaviours, ranging from aggression to emotional perception, in a sex-specific manner [11,18]. In males, androstadienone plays a role in modulating responses to competitive situations by enhancing the saliency of potential threats. Studies have shown that exposure to androstadienone reduces interference in the visual processing of threatening facial expressions in males, suggesting a preparatory mechanism for conflict [19]. This explanation is in line with findings that androstadienone serves as a dominance-conveying chemosignal, which consequently induces submissive responses and cooperative behaviour among males during competitive interactions [12,14]. Androstadienone therefore contributes to increased cooperation and reduced aggression among males. In contrast, androstadienone’s influence on females may be the opposite. Parma *et al*. [20] found that after exposure to androstadienone, females at low conception risk rated female faces as less attractive. The authors attributed this to a competitive mindset induced by androstadienone, resulting in an increased propensity for competition strategies [20]. Regarding prosocial behaviour, this induced competitiveness could reduce generosity, as individuals exhibited behaviour more frequently that emphasized their own interests rather than egalitarianism or altruism [21]. Furthermore, a recent study showed that females smelling androstadienone demonstrated increased reactive aggression [11], a behaviour known to be negatively associated with prosociality [22]. Ultimately, the intriguing contrast between the effects of androstadienone on males and females underpins our hypothesis that androstadienone may increase prosociality in males while reducing it in females.

In the current study, we employed a social discounting task [23] to measure generosity, a particular form of prosociality. The task differs from other tasks in which prosocial motives and other social motives, such as reputation building, strategic responding and status management, are frequently confounded [24,25]. Thus, the task provides an opportunity to isolate and investigate the effects of androstadienone on prosocial behaviour in a more refined and precise manner. Normally, the level of generosity tends to decrease as the level of intimacy decreases [26]. However, alternative patterns are also possible, as prosocial behaviour may not be uniformly influenced across all social distances under certain conditions. For example, rather than reducing generosity in a general manner, testosterone decreases altruism only in males and only towards distant others [25,27]. Based on these considerations, we reasoned that androstadienone could either impact overall prosociality or differentially influence prosociality between close and distant others in a sex-specific manner. We also explored two potential underlying mechanisms. Specifically, androstadienone might influence the initial bias towards the generous option in a social discounting task or it might affect perceived social distance towards others that consequently shapes prosociality. Here, we employed a drift-diffusion model (DDM) approach to address this question.

## 2. Methods

### 2.1. Participants

Following our pre-registration, we recruited 170 participants (98 females; mean age = 22.67, s.d. = 2.97, range = 18–34) through mass email and posters on campus. All the participants were Han Chinese, heterosexual (Kinsey score = 0), had a normal or corrected-to-normal vision, a normal sense of smell assessed by self-report and no respiratory allergy or upper respiratory infection. None of the female participants had used contraceptive pills over the past three months. The sample size was based on the effect size reported in our previous study, which tested the effects of androstadienone on human aggression [11]. The required sample size was determined by G^∗^Power 3.1 [28] to detect a medium effect size (Cohen’s *f* = 0.241) with a power of 0.80 (*a* = 0.05). The *a priori* analysis resulted in a sample size of 137 (ANOVA: fixed effects, special, main effects and interactions). Nevertheless, we decided to recruit 170 participants to allow for possible non-compliance or impossibility of model fit. A total sample of 170 would ensure 137 compliant samples. In addition, as described in §3, seven participants were excluded from data analyses due to their consistent differentiation of androstadienone from control stimuli. This resulted in a final sample size of 163 participants (92 females; mean age = 22.60, s.d. = 2.92, range = 18–34).

Female participants were asked to estimate the period of their menstrual cycle based on previous records and were invited to the experiment during the periovulatory phase (e.g. around the midpoint of the menstrual cycle).

The study was approved by the Institutional Review Board of Hong Kong Polytechnic University and conducted in accordance with the Declaration of Helsinki for Medical Research involving Human Subjects. Participants were informed about the study and provided written consent before the experiment. Following completion of all tasks, participants were compensated 105 HKD as a flat fee plus a variable amount generated by a computer depending on their decisions during the social discounting task. The total amount ranged from 111.5 to 114.5 HKD (*M* = 112.65, s.d. = 1.16).

### 2.2. Olfactory stimuli

Our study employed olfactory stimuli that included androstadienone (at a concentration of 500 μM in a solution containing 1% v/v clove oil and propylene glycol) and a control solution consisting of 1% v/v clove oil in propylene glycol. The androstadienone concentration selected for this research (500 μM) followed the common practice of studies in this field [6,11,29]. Active and control stimuli were offered to participants in identical 40 ml polypropylene jars containing 5 ml of the solution. The jars were connected to two Teflon nosepieces using a Y-structure. The distinguishability of the two olfactory stimuli was assessed near the end of the experiment using a triangle odour discrimination task. Each participant completed six trials in the odour discrimination task. In each trial, three scents (two bottles with identical control solution and one bottle with androstadienone solution) were presented. Participants were asked to identify the scent that differed from the other two after being exposed to all three scents while blindfolded. The chance of correctly identifying the odd scent by chance was one in three.

### 2.3. Procedures

Upon arrival, participants provided their basic demographic information and reported their olfactory sensitivity on a 4-point scale, ranging from 1 (good) to 4 (bad). They were also given a series of questionnaires to complete, serving as measures for our exploratory analysis where potential interactions between these variables and the androstadienone intervention were assessed. The questionnaire battery included the Interpersonal Reactivity Inventory (IRI) [30], which measures the respondent’s capacity for empathy; the Social Value Orientation (SVO) [31] questionnaire, which assesses the participant’s preferences in allocating resources between themselves and others; and a questionnaire gauging their subjective socioeconomic status [32]. To investigate changes in emotional states related to exposure to the olfactory stimuli, participants also filled out the state version of the Positive and Negative Affect Schedule (PANAS) [33], both before and after the olfactory exposure.

After filling out the PANAS scale for the first time, participants were randomly assigned to either the androstadienone group or the control group in a double-blind, placebo-controlled, between-participant design. The androstadienone group consisted of 41 females and 42 males, and the control group comprised 51 females and 29 males. Overall, there were 92 female (mean age = 22.80, s.d. = 2.82, range = 18–34; 30.6% bachelor’s, 56.1% master’s and 13.3% doctoral) and 71 male participants (mean age = 22.40, s.d. = 3.18, range = 18–32; 40.3% bachelor’s, 36.1% master’s and 23.6% doctoral). Then, they were provided with a jar containing either the target or control stimuli, which was linked to two Teflon nosepieces using a Y-structure. Participants held the jar with their non-dominant hand, placed the nosepieces inside their nostrils, and were asked to breathe in through their nose and out through their mouth for the duration of subsequent tasks. While being exposed to their assigned olfactory stimuli, participants completed the measurement of social distance perception [26], which characterizes closeness to a specific person. Participants rated their level of closeness to the following target individuals on a 20-point Likert scale, ranging from 1 (very close) to 20 (not close): mother, father, sibling, partner, child, grandparent, family member, kin, best friend, member of circle of friends, colleague, neighbour, acquaintance and stranger. Once finished, participants were briefed on the social discounting task [23]. The social discounting task lasted about 8 min and was carried out in a controlled setting with the experimenter in a separate, adjacent room, observing the participants through a monitor. To prevent cross-contamination, the same type of stimulus was administered to all participants during a single day of testing.

Following the social discounting task, each participant completed the triangle odour discrimination task mentioned above. They also rated the pleasantness and intensity of the target and control stimuli using a 9-point Likert scale (1 = not at all pleasant/intense; 9 = extremely pleasant/intense). This session took around 3 min.

### 2.4. Social discounting task

Participants underwent an assessment of social distance perception before engaging in the main task. This measurement aimed to familiarize participants with social distance in the social discounting task by associating different individuals in the social environment (e.g. varying social distances) with different levels of closeness. Lower closeness ratings (e.g. 1 = very close) implied shorter perceived social distance. The measurement of social distance perception occurred during exposure to olfactory stimuli. Given that perceived social distance underpins generosity, this measurement may elucidate the mechanism underlying the effect of androstadienone (e.g. a potential mediation effect of perceived social distance).

Prosociality was measured using an adapted version of the social discounting task [26]. The task was programmed using PsychoPy [34]. Participants identified individuals at specific social distances: 1, 2, 3, 5, 10, 20, 50, and 100 in their social environment. They provided the names and contact details of these individuals for the possible distribution of monetary awards, excepting strangers at social distances 50 and 100. Participants were asked to include only individuals with whom they shared positive or neutral relationships. During the task, participants repeatedly chose between a selfish alternative (receiving a varying amount of money that was larger than or equal to the amount they received with the generous alternative) and a generous alternative (receiving a fixed amount, with the same amount given to the person at one of the eight social distances). The selfish alternative ranged from 130 HKD to 290 HKD in increments of 20 HKD. The generous alternative involved both the participant and the person at the designated social distance each receiving a constant sum of 130 HKD. Each combination of social distance and selfish amount was presented once, making a total of 72 unique trials (8 distances by 9 selfish amounts). Participants had to respond within 6 s, otherwise, the trial was skipped. The skipped trial was re-presented one more time at the end of the current social distance level. Both the order of the blocks representing different social distances and the trials within each block were randomized.

Our task was incentive-compatible (ensuring that decisions reflected actual preferences). One trial was randomly selected, and 5% of the chosen amount(s) were paid out at the end of the experiment, either to the participant alone or also to the individual specified by the participant for the social distance of the selected trial.

**Figure 1.**
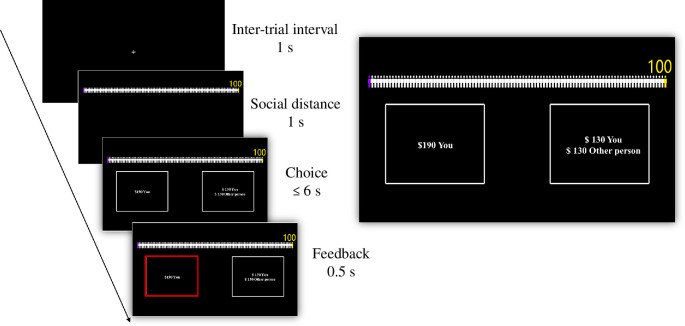
Social discounting task.

A graphical depiction of the social discounting task is shown in figure 1. Every trial incorporated both numeric and iconographic displays to represent social distance. Distances were indicated numerically (1, 2, 3, 5, 10, 20, 50 or 100), and two distinct icons visually represented the participant and the partner (a purple icon representing the participant, a yellow one representing the partner). The spatial gap between these icons dynamically mirrored the indicated social distance in each trial. The positions of the two options on the left or right of the display were randomized to eliminate any positional bias. Participants had up to 6 s to make their decision and the chosen option was highlighted by a red rectangular frame for 0.5 s to indicate that the response was registered. Trials were separated by a 1 s inter-trial interval, in which participants were presented with a fixation cross to prepare for the next trial.

### 2.5. Statistical analysis

The statistical analysis of this study consisted of multiple steps. Initially, the impact of the olfactory stimuli was assessed, particularly focusing on the changes in affect brought about by exposure to the odour.

Next, as a preliminary analysis, a generalized linear mixed model (GLMM) was used to analyse the binary choice and log-transformed response times in the social discounting tasks. The model incorporated condition, sex and their interaction, along with social distance, as fixed effects. Participants were considered as a random effect, allowing for a random intercept for the model. Considering the effect of social distance perception could vary across participants, we also added a by-participant random slope for social distance.

In our main analysis, we incorporated both model-free and model-based methods to examine the effect of androstadienone on prosocial behaviour. This allowed us to gain convergent evidence from different parametrizations, enhancing the robustness of our conclusions. In the model-free approach, no assumptions were made about the shape of the discount function, thus providing an evaluation of generosity without strong assumptions, based on the area under the curve (AUC) [35]. In our study, we evaluated the AUC corresponding to the amount forgone at each social distance across all groups. For each participant, the amount forgone at each social distance was estimated based on logistic regression, with the choice as the dependent variable and the selfish reward amount as the independent variable. Specifically, the amount forgone was the amount of money at which the participant chose the selfish alternative with 50% probability (i.e. the indifference point). If a participant exclusively chose one option (e.g. the selfish alternative), the amount forgone was set at half of an increment below and above the range of selfish options, resulting in 120 HKD and 300 HKD, respectively. Then, 130 HKD was subtracted from each indifference point to determine the cost of being generous (i.e. the amount forgone). We derived the AUC for each individual by normalizing the amount forgone as a fraction of its maximum value and the social distance in terms of its maximum value. These normalized points were linked with straight lines, leading to the formation of trapezoids that were then summed up [36]. After these standardization steps, the AUC could theoretically range from 0 (highest possible level of discounting) to 1 (no discounting as social distance increases).

The model-based approach, on the other hand, involved fitting the data to a hyperbolic function [23]


(2.1) v=V/(1+kD)

where *v* was the amount forgone at specific social distance *D*, *V* represented the willingness to be generous at *D* = 0 and *k* was the discount rate. This model allowed us to quantify the willingness to be generous at a close social distance (*V*) and how rapidly generosity declined as social distance increased (*k*).

Both the model-free and model-based methods were implemented as per our pre-registration. However, both approaches were sensitive to the estimated indifference points, which were based on logistic regressions. Therefore, estimates based on these methods were sensitive to outliers and unsystematic data points, which indeed occurred in our samples. To preclude this potential methodological bias, we used a third approach that was not pre-registered: maximum likelihood estimation (MLE). MLE does not require all of the 72 trials to conduct parameter estimation. To control for data quality, we deleted trials with response times shorter than 300 ms. Under the softmax selection rule, the likelihood function of a single trial is defined as


(2.2) $$p\left(k,V,\sigma \right)=\left\{ \begin{array}{l} \frac{1}{1+e^{-\sigma \left(Value_{selfish}-Value_{generous}\right)}},if\;the\;selfish\;option\;is\;selected\\ 1-\frac{1}{1+e^{-\sigma \left(Value_{selfish}-Value_{generous}\right)}},otherwise, \end{array}\right.$$

where *p* is the probability of choosing a particular option, σ measures to what extent decisions are guided by the value differences and the value represents the subjective value of the selfish or generous option. For selfish alternatives, the value is the amount of money of the option. For generous options, the value is


(2.3) $$Value_{generous}=130+\frac{V}{1+kD}$$

We log-transformed the *k* parameter before our formal analysis.

We then investigated potential hormonal effects by assessing group differences in social distance perception. We calculated a mixed ANOVA to investigate the effect of the target individual (e.g. mother), sex and androstadienone administration on self-report closeness to the target individuals.

We also modelled response times and choice data using the DDM [37]. The DDM is a sequential sampling model that simultaneously accounts for choices and response times. The model assumes that decision-making between two alternatives is inherently biased (*β*) towards one option. This bias is combined with the evidence provided by the choice options. As decision-makers consider the two options, the evidence gradually accumulates and the speed with which this happens is captured with a drift rate (*δ*). The evidence in favour of one of the two options ultimately reaches a threshold that quantifies the level of evidence required to make a decision (*α*). The model posits that the time it takes to reach this threshold (partly) explains response time, in which an additional non-decision process is represented by *τ*. The lower bound for reaction time across all participants was set to 0.1 s, referring to the hBayesDM R package [38]. The DDM provides a more detailed examination of the decision-making process, enabling a nuanced understanding of the influence of androstadienone on social discounting behaviour. We estimated posterior distributions of the four parameters for each participant under the hierarchical Bayesian framework [39]. We created predictors capturing the effect of sex, condition and their interactions on each of the four parameters, resulting in 12 parameters. For example, the group-level threshold parameter can be represented as


(2.4) $$\alpha =\mu +\beta_{sex}sex+\beta_{condition}condition+\beta_{sex\;by\;condition}interaction$$

where *μ* is the grand mean and *β* characterizes the group difference of interest (e.g. sex differences). The priors for these parameters were specified by standard normal distribution. Bayesian inference and optimization were conducted using the Stan programming language [40]. We estimated the posterior distribution of parameters by using Markov chain Monte Carlo (MCMC) methods. Four chains of 4000 iterations were run, with half of the iterations serving as warm-up. The convergence of the MCMC chains was evaluated based on the R-hat convergence diagnostic [41], which should be less than 1.01 to indicate the chains have mixed well. We concluded a credible group difference if the 95% highest density interval (HDI) did not contain zero [39]. Similar to MLE, we also excluded trials faster than 300 ms.

## 3. Results

### 3.1. Olfactory stimuli and odour discrimination task

To investigate whether participants’ affect was influenced by olfactory stimuli, we conducted a mixed ANOVA with olfactory condition and sex as between-subject factors and time (pre- versus post-exposure) and valence (positive versus negative affect) as within-subject factors on PANAS ratings. There was a significant two-way interaction between time and valence, indicating that positive affect dropped more strongly than negative affect over time, *F*(1, 166) = 22.13, *p* < 0.001, *η*_g_^2^ = 0.01. In addition, there was a significant main effect of sex on ratings, *F*(1, 166) = 13.66, *p* < 0.001, *η*_g_^2^ = 0.04. Females exhibited generally lower levels of both positive and negative affect than males.

Regarding the odour discrimination task, participants discriminated the olfactory stimuli with better than chance accuracy (mean ± s.d. = 0.40 ± 0.25 versus chance (0.33), *t*(169) = 3.34, *p* = 0.001, Cohen’s *d* = 0.26). Perceived intensity was significantly higher for the control stimuli, *t*(169) = −3.78, *p* < 0.001, Cohen’s *d* = −0.29. There was no difference in perceived pleasantness, *t*(169) = −0.60, *p* = 0.55. Seven of the 170 participants correctly identified the target in all six trials. We excluded these seven individuals from subsequent data analysis, as their ability to correctly identify the target odour in all the trials could potentially indicate a heightened sensitivity or awareness of the olfactory stimuli, which might have confounded the intervention effects.

Next, we conducted a 2 (sex: males versus females) by 2 (condition: androstadienone versus placebo) ANOVA on the odour sensitivity score. There was a significant main effect of sex, *F*(1, 159) = 4.54, *p* = 0.035, *η*_g_^2^ = 0.03. In comparison with males, females reported lower subjective olfactory sensitivity. We found no main effect of sex, condition and their interaction on accuracy in the odour discrimination task (all *p*s > 0.28). For example, the sex difference in subjective olfactory sensitivity was not reflected by objectively different accuracy in the olfactory discrimination task *F*(1, 159) < 0.01, *p* = 0.96, *η*_g_^2^ < 0.01.

### 3.2. Preliminary analysis: choice and response time

For the choice data, results of the generalized linear mixed model revealed a significant fixed effect for social distance, *b* = 0.045, *z* = 17.94, *p* < 0.001, indicating that as social distance increased, participants were more likely to choose the selfish option. This suggests that participants processed social distance. Neither the condition, sex nor the interaction between condition and sex were found to significantly affect choice (all *p*s > 0.053). Given the significant change in positive affect over time, we also added the change of positive affect as a covariate. The statistical significance of the fixed effects remained unchanged (all *p*s > 0.054).

For response time data, the linear mixed model did not reveal any significant effect of sex, condition, interaction and social distance (all *p*s > 0.21). Thus, participants across sexes and groups spent comparable amounts of time on decisions at different social distances.

### 3.3. Androstadienone and social discounting

A summary of model-free and model-based estimates is shown in table 1. Following the hypothesis of our pre-registration, we first tested whether androstadienone administration had a sex-specific effect on AUC, our model-free measure of prosociality. We performed a between-subject ANOVA with group and sex as between-subject factors. The interaction between group and sex was non-significant, *F*(1, 143) = 1.41, *p* = 0.24, *η*_g_^2^ = 0.01. The androstadienone group did not differ significantly from the placebo group in AUC, *F*(1, 143) = 0.81, *p* = 0.37, *η*_g_^2^ = 0.01, neither was there a sex effect, *F*(1, 143) = 0.70, *p* = 0.41, *η*_g_^2^ < 0.01. Thus, androstadienone did not affect generosity measured by the social discounting task.

**Table 1.** Social discounting task parameters by condition and sex. The values given in the table are means with s.d. in brackets.

estimates	androstadienone		placebo	
	females	males	females	males
AUC	0.35 (0.24)	0.34 (0.28)	0.27 (0.21)	0.36 (0.28)
*k*	0.11 (0.13)	0.20 (0.41)	0.30 (0.81)	0.16 (0.34)
*V*	162 (79.5)	175 (78)	182 (140)	174 (72)
*k* (MLE)	0.18 (0.19)	0.24 (0.26)	0.21 (0.25)	0.22 (0.27)
*V* (MLE)	268 (146)	295 (153)	268 (160)	288 (144)

To further interrogate our data, we examined whether androstadienone influenced the shape of the social discounting function. We fitted a standard hyperbolic discounting model to the amount forgone for each participant individually. First, we conducted an ANOVA to examine the interaction effect between sex and group on the discount rate (*k*). There was no significant interaction effect, *F*(1, 119) = 0.03, *p* = 0.86, *η*_g_^2^ < 0.01, nor significant main effects (both *p*s > 0.55). Second, we examined whether androstadienone administration influenced the intercept of the hyperbolic function (*V*). The two-way ANOVA did not reveal any significant effect, with all *p*s > 0.53.

**Figure 2.**
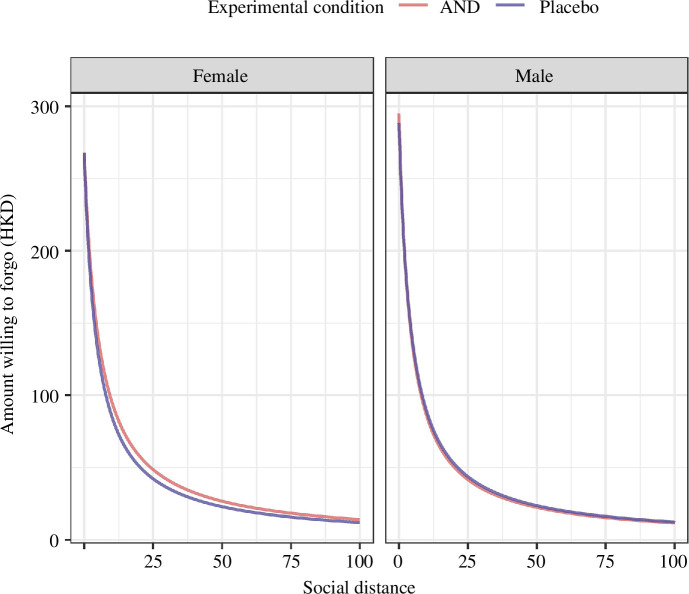
Effects of androstadienone administration on prosociality based on MLE. The amount forgone as a function of social distance is estimated by MLE. The lines represent the group-level hyperbolic function whose parameters are based on the mean of individual-level parameters. AND = androstadienone.

The non-significant effect of androstadienone was corroborated by MLE analyses (figure 2). We implemented an ANOVA to examine the interaction effect between sex and group on social discounting parameters derived from MLE. The results revealed no significant interactions or main effects of sex and experimental conditions on social discounting (all *p*s > 0.34). Similarly, there was no significant effect on the intercept *V* (all *p*s > 0.32).

### 3.4. Androstadienone’s effects on social distance perception

Given the non-significant effect of androstadienone administration on prosociality, we explored whether the administration affected social distance perception. We conducted a three-way mixed ANOVA with sex and condition as between-subject factors and the target individual as a within-subject factor. There were no significant three-way or two-way interactions. The results revealed a significant main effect of the target individual, *F*(7.11, 667.89) = 220.21, *p* < 0.001, *η*_g_^2^ = 0.61, indicating that social distance perception varied significantly across target individuals. In addition, females exhibited generally higher closeness ratings and thus greater social distance to the target individuals than males, *F*(1, 94) = 10.85, *p* = 0.003, *η*_g_^2^ = 0.04.

### 3.5. Drift-diffusion model

**Figure 3.**
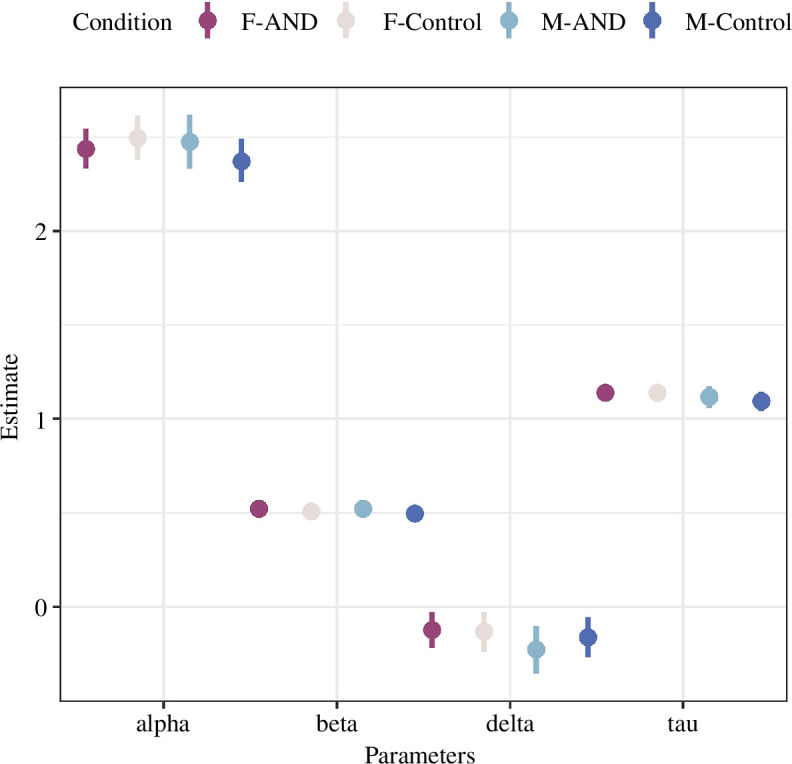
The estimates of the DDM. Posterior modes (dots) and 95% highest density interval (lines) for the four parameters for each of the four conditions. F = females; M = males; AND = androstadienone; Control = placebo.

All individual- and group-level parameters showed an R-hat convergence diagnostic smaller than 1.01, indicating that the four MCMC chains converged and the estimates were reliable. We extracted the 12 dummy coded parameters to check whether there was any meaningful group difference across the four conditions. None of the 95% HDI of these parameters deviated from zero. Therefore, we were unable to conclude any credible group difference in decision boundary, initial bias, drift rate and non-decision time. Based on this result, we computed posterior modes for each of the four conditions and plotted their 95% HDI (figure 3).

### 3.6. Exploratory analysis of individual differences

To investigate whether the non-significant effect of androstadienone was confounded by other contributing factors, we compared group differences in the four factors of the Interpersonal Reactivity Inventory (perspective taking, fantasy, empathic concern and personal distress), the Social Value Orientation and the subjective socioeconomic status across the two experimental groups in each sex. For females, there were no significant differences in these factors between olfactory conditions (all *p*s > 0.12). For males, marginally significant differences were observed for personal distress, *t*(62.7) = 1.96, *p* = 0.054, Cohen’s *d* = 0.47, and perspective taking, *t*(63.3) = 1.97, *p* = 0.054, Cohen’s *d* = 0.47. Therefore, we included these two factors as predictors in two linear regression models to predict the slope (*k*) and intercept (*V*) based on MLE. Personal distress significantly predicted social discounting (*k*), *b* = 0.085, *t* = 2.40, *p* = 0.018. No other significant results were observed. Therefore, the null effect of androstadienone cannot be attributed to the measured individual differences.

## 4. Discussion

In the pursuit of understanding the nuanced role of human chemosignals, the current study examined the effects of androstadienone on a specific type of prosocial behaviour, social distance-dependent generosity. As per our pre-registration, we predicted that the influence of androstadienone would be sex-specific, with males exhibiting increased generosity and females exhibiting more selfish responses. Despite our initial hypothesis and some supporting prior literature, we found no significant shift in prosocial behaviour across sexes under the influence of androstadienone. This result is at odds with previous reports that androstadienone influences higher cognitive processes like generosity [5], warranting a closer examination of the underlying mechanisms. Our secondary analysis found neither significant influence of androstadienone on perceived social distance nor meaningful effects on the four main parameters of the DDM. In the following section, we discuss our results in the context of the current literature.

Early investigations of androstadienone are marked by its potential influence on emotions and physiological arousal, given the anatomical overlap between the olfactory system and limbic system related to emotional responses [42]. Indeed, it has been well established that androstadienone positively modulates psychophysiological arousal and mood [8,9]. For example, exposure to a relatively low concentration of androstadienone increased the processing of stimuli with emotional significance [10] and positive mood while decreasing negative mood, especially among females interacting with a male experimenter [43]. Unexpectedly, our study reported a decrease in positive affect among participants exposed to androstadienone, regardless of sex. This inconsistency with previous studies prompted us to examine odour sensitivity across sexes and conditions, as the different patterns in affective arousal may derive from different odour sensitivity between study samples, which consequently could have introduced different levels of perceptual bias [5]. Although female participants rated themselves lower in the odour sensitivity relative to male participants, this discrepancy was not reflected in the accuracy of the odour discrimination task. In addition, we excluded participants who could perfectly discriminate between androstadienone and the placebo in all six trials. Therefore, it is unlikely that the sex differences in odour sensitivity affected subsequent behavioural results. Nevertheless, the discrepant affective responses in the current research compared with previous research should be further discussed in conjunction with the effect of androstadienone on prosocial behaviour.

Our main finding, i.e. that androstadienone does not influence prosocial behaviour, contrasts with empirical evidence that both female [5] and male participants [12] were more generous in the presence of androstadienone. Specifically, our analysis based on AUC and parameters of the hyperbolic function suggested that androstadienone does not induce a general shift in altruism, nor drive parochial altruism [44], i.e. increased generosity towards close others and decreased generosity towards distant others. Apart from the difference between measures of prosociality, there are several potential reasons for this null effect.

First, failure to increase positive emotion may explain why androstadienone had little effect on generosity, at least in females. It has been found that androstadienone exposure promoted both attractiveness ratings of potential mates and mood among females [45]. Therefore, the general positivity of affective dispositions induced by androstadienone may be responsible for its sex-specific effects on cognition and behaviour [5,46]. Indeed, a strong correlation between generosity and positive mood increment was identified among females exposed to androstadienone [5]. By contrast, merely 44 of our 170 (25.88%) participants reported an increase in positive emotions. Nevertheless, empirical studies have also found higher perceived attractiveness of targets in the absence of increased positive mood [46], suggesting an additive rather than necessary influence of positive mood [5]. Indeed, our supplemental analysis, focusing on the intervention effect among participants who experienced elevated positive emotions, did not find any significant main or interaction effects (all *p*s > 0.081), and thus failed to provide evidence for the necessary influence of positive mood.

Second, the effect of androstadienone could be context-dependent. Theoretical frameworks in behavioural endocrinology suggest that the evolved function of hormonal signals is the coordination of diverse behavioural responses to specific eliciting conditions [47]. It has been found that testosterone could induce parochial altruism, but only in the context of intergroup competition [27]. This finding challenges the common perception that testosterone invariably encourages antisocial and aggressive behaviours, instead highlighting its particular function in fine-tuning social cognition. This line of reasoning also applies to the current research question. For example, the positive mood change in females exposed to androstadienone was only identified when the experiment was conducted by an experimenter of the opposite sex [43]. Moreover, male generosity may constitute a mating signal, as males exhibited greater generosity in the presence of an attractive female compared with a same-sex observer or no observer at all [48]. Therefore, the absence of a relevant context or eliciting condition in which chemosignals exert their influences might explain the non-significant effects of androstadienone on prosocial behaviour.

Furthermore, previous studies have highlighted the significance of the sex of both dictator and recipient in determining the donation amounts in a dictator game [49]. Therefore, a third factor that may block the effect of androstadienone on generosity is the sex of the target (e.g. recipient). Androstadienone has been hypothesized to be associated with mating and sexual behaviour [50] given its influence in conveying information about mate quality and increasing the perceived attractiveness of potential mates [15]. Indeed, evidence has shown that the positive effect of androstadienone on attractiveness ratings is found for the opposite sex only [46]. Considering that perceived attractiveness shapes cooperative and prosocial behaviour [17], it may be important to distinguish the sex of targets while investigating generosity. However, research on the role of androstadienone in mate selection and perceived attractiveness has equivocal findings [51]. To fully address the potential influence of the sex of the target in prosocial behaviour, further empirical evidence is required.

While conflicting with our hypothesis, our findings resonate with previous studies that failed to establish androstadienone as a human pheromone when investigating its effect on perceived attractiveness of opposite-sex faces [46,52] or mood [53]. Indeed, the evidence for androstadienone as a putative human pheromone has remained elusive, with studies struggling to obtain robust and replicable results [54]. Positive findings need to be treated with scepticism, as the literature is plagued by inconsistencies and potential methodological limitations [55]. Our study adds to the growing body of evidence casting doubt on the pheromonal properties of androstadienone, at least in the context of social decision-making. These collective findings underscore the need for rigorous and well-controlled investigations to determine the role of androstadienone in human chemosensory communication and social behaviours.

Several limitations of our study should be noted. First, the recruited participants all came from the university population in Hong Kong. This demographic choice may restrict the generalizability of our findings to broader populations. For example, we find no significant effect of sex on prosociality, contrasting with previous findings showing that males were less generous than females toward people they felt closest to [56]. However, findings regarding the effect of sex on prosociality are equivocal [57]. It is also possible that the substantial individual differences of prosociality overshadow or bias potential sex differences, especially if the sex difference is subtle. Second, the current study used only the social discounting task to measure generosity. Future research should incorporate more diverse measures of prosocial behaviour and account for both the donor’s and the recipient’s sex to enable a more accurate appraisal of androstadienone’s impact.

In conclusion, our findings suggest that the influence of androstadienone on prosocial behaviour may not be as straightforward as predicted. The inconsistencies between our findings and previous literature underscore the importance of further explorations into the effect of androstadienone on social behaviour, including the potential influences of effect, arousal, relevant social context and sex.

## Data Availability

The raw data, materials, R, and Stan code are available at [58]. This study was pre-registered at Aspredicted (https://aspredicted.org/see_one.php). Electronic supplementary material is available online at [59].
